# Diabetes mellitus and pregnancy in Wolfram syndrome type 1: a case report with review of clinical and pathophysiological aspects

**DOI:** 10.3389/fmed.2025.1639884

**Published:** 2025-09-08

**Authors:** Amelia Caretto, Francesca Scarascia Mugnozza, Fanny Valsecchi, Erika Pedone, Mirko Pozzoni, Susanna Rosa, Andrea Laurenzi, Giulio Frontino, Raniero Chimienti, Lorenzo Piemonti, Marina Scavini

**Affiliations:** ^1^Diabetes Research Institute, IRCCS San Raffaele Scientific Institute, Milan, Italy; ^2^Vita-Salute San Raffaele University, Milan, Italy; ^3^Department of Obstetrics and Gynaecology, IRCCS San Raffaele Scientific Institute, Milan, Italy; ^4^Department of Pediatrics, IRCCS San Raffaele Scientific Institute, Milan, Italy

**Keywords:** Wolfram syndrome, fertility, pregnancy, pregestational diabetes, automated insulin delivery, rare disease, therapy

## Abstract

Wolfram syndrome type 1 (WS1) is a rare genetic disorder characterized primarily by non-autoimmune diabetes mellitus, optic atrophy, deafness, and diabetes insipidus. It may include other endocrine, urological, psychiatric, and neurological disorders. The syndrome arises from mutations in the *WFS1* gene, which encodes the Wolframin protein, a key regulator of endoplasmic reticulum (ER) function in pancreatic beta-cells and other tissues. Diabetes in WS1 typically has an early-onset, progresses slowly, and is characterized by insulin deficiency, low insulin requirement, and a lower incidence of chronic complications compared to type 1 autoimmune diabetes. Nowadays, there is no cure for WS1, and management relies on the treatment of the different associated conditions. Fertility can be compromised due to hypogonadism, although cases of successful pregnancy have been reported. These are high-risk pregnancies due not only to hyperglycemia, but also to the other comorbidities of the WS1. This review discusses the peculiarities of diabetes associated with WS1 and the reproductive outcomes in WS1, reporting a case of successful pregnancy in a woman with WS1 treated with a hybrid closed-loop insulin pump.

## 1 Introduction

Wolfram syndrome (WS) is a rare genetic disorder characterized by the presence of diabetes mellitus, optic atrophy, deafness, and diabetes insipidus, also known as DIDMOAD (1, 2). It is a neurodegenerative progressive disorder, transmitted in an autosomal recessive manner, which may include various pathological conditions (3). Its prevalence can vary across different geographical areas, from 1 in 100,000 people in North America to 1 in 770,000 in the UK (4–6). In Italy, the prevalence of WS is estimated to be 1 in 1,351,000, with peaks of prevalence in certain regions (i.e., 1 in 54,478 in North-Eastern Sicily) (7, 8).

Depending on the mutant gene, there are two types of WS (OMIM 222300). WS type 1 (WS1) is caused by a mutation in the *WFS1* gene or *Wolframin gene*, located on chromosome 4p16.1, encoding the Wolframin protein, which is involved in the regulation of the endoplasmic reticulum (ER)’s unfolded protein response (UPR) within the pancreatic beta-cells and other tissues (9). WS type 2 (WS2) is caused by a mutation in the *CISD2* gene on chromosome 4q22, encoding the protein ERIS (ER intermembrane small protein), also associated with the ER (10). Furthermore, there are also forms of autosomal-dominant inheritance associated with heterozygous pathogenic variants of *WFS1* that are collectively termed “WFS1-related disorders” (11).

Diagnosis of WS1 is often difficult because symptoms appear subtly at different times in the patient’s life, with genetic testing often being delayed, postponing correct diagnosis and appropriate treatment. Diabetes mellitus is often one of the earlier manifestations. Prognosis of the disease usually depends on the severity of neurological symptoms, which may lead to premature death between the ages of 25–50 years of age (6, 11). Clinical features of WS1 are the following: diabetes mellitus, optic atrophy, diabetes insipidus, sensorineural hearing loss, neurological disorders (e.g., brain atrophy, ataxia, central apnea), urinary tract dysfunctions (incontinence, neurogenic bladder, urinary tract infections), hypogonadism, central hypothyroidism, growth retardation, psychiatric illness, gastrointestinal issues (11).

In this review, we aim to explore the peculiarities of diabetes in WS1 and how it affects fertility and pregnancy, reporting a case of a woman with WS1 who safely delivered after spontaneous conception.

## 2 Diabetes in Wolfram syndrome

Diabetes mellitus (DM) in WS1 is a non-autoimmune diabetes with onset in youth, characterized by the loss of beta-cells, with low C-peptide levels. Although rare, some cases of positivity of autoantibodies specific for type 1 diabetes (T1DM) in WS1 are reported in the literature (12). DM is often one of the first manifestations of the syndrome, as it usually occurs within the age of 16 years, usually around 6 years of age (13). The onset of diabetes is most of the time slow and without ketoacidosis, insulin requirement is low, and microvascular and macrovascular complications are rare (13–15). DM in WS1 is caused by a dysfunction of ER management that negatively affects beta-cell mass maintenance.

### 2.1 WS as a model of ER distress disease

The protein Wolframin is an ER transmembrane protein involved in regulating ER calcium homeostasis, the UPR, and vesicular transport from the ER to the Golgi apparatus (16–19). ER is an intracellular organelle fundamental for post-translational modification and folding of proteins. Proteins correctly folded will progress down the secretory pathway, while not correctly folded proteins will be dismissed by a specific ER-associated pathway (20). When the ER load overcomes its folding capacity (i.e., viral infections, environmental toxins, inflammatory cytokines, decreased ER calcium levels, mutant protein), the ER enters a condition of stress. Mutations in the coding region of highly expressed proteins, handled by the ER, may directly perturb their folding and cause ER stress (21). Cells face ER stress by triggering the UPR that has three major regulators: activating transcription factor 6α (ATF6α), protein kinase R-like ER kinase (PERK), and inositol-requiring enzyme 1α (IRE1α) (21, 22).

WFS1 protein has a fundamental role in preserving beta-cell function and viability in physiologic and pathophysiologic states through the positive regulation of insulin production and the negative regulation of ER stress-inducible pro-apoptotic factors.

WFS1 deficiency causes dysregulation of insulin production and secretion, ER calcium depletion, and calpain 2 activation, resulting in activation of an apoptotic pathway (23).

In 2020, Abreu et al. (24) demonstrated that in *WFS1* knockout mice, there is ER stress, beta-cell dysfunction, and impaired insulin secretion that leading to a progressive decline in glucose control and insulin deficiency. The abnormal islet architecture in that animal model reflects the cellular dysfunction seen in patients with WS1. *WFS1* loss causes a reduction of beta-cell number and also affects beta-cell maturity, leading to an abnormal beta/alpha cell ratio in the pancreatic islets. The authors also reveal that under physiologic conditions, WFS1 promotes insulin production and modulates pro-apoptotic molecules of the ER stress response to sustain an adaptive response through AKT activation (24). Under pathologic conditions, as in the case of a mutated *WFS1*, the absence of WFS1 leads to unregulated ER stress that culminates in beta-cell death, at least partly due to the repression of AKT survival pathways. Moreover, WFS1 is likely to be involved also in type 2 diabetes mellitus (T2DM), as islets from T2DM donors had significantly lower levels of *WFS1* and insulin gene expression than islets from donors without diabetes (24).

According to a recent paper by Amo-Shiinoki et al. (25), the endocrine dysfunction in WS is due to beta-cell dedifferentiation and loss of cellular identity. An immunohistochemical analysis of human pancreatic sections from deceased donors with WS1 highlights a significant loss of beta cells and consequent decrease in alpha cells. Authors found that inhibition of thioredoxin-interacting protein (Txnip), a protein involved in metabolic stress response, preserved functional beta-cells and improved metabolic dysfunction in a *WFS1*-knockout mouse model of WS1 (25). Also, beta cell inflammation seems to have a role in the pathogenesis of diabetes in WS1. The inflammation induced by excessive ER stress is called sterile inflammation, as it is not associated with infections. The UPR pathway interacts with IkB, an inhibitor of the nuclear factor-κB (NFκB), which is a family of transcription factors regulating gene expression of pro-inflammatory cytokines, and regulates the nuclear translocation of NFkB. These mechanisms may lead to sterile inflammation (26, 27). As beta cells have a high load for ER, they are prone to ER stress and consequently sterile inflammation (28). Morikawa et al. (29) found islet-localized inflammation in WS1 models and suggested that WFS1 works to regulate sterile inflammation in pancreatic beta cells. PERK and IRE1α pathways mediate high glucose-induced inflammation in a beta cell model of WS1, while macrophage infiltration and hypervascularization were detected in the islets of *WFS1*-knockout mice (29).

In addition to its well-established role in ER stress regulation, Wolframin is a critical modulator of mitochondria-associated ER membranes (MAMs) and mitochondrial function. ER stress have an impact on mitochondrial dynamics through inositol 1,4,5-trisphosphate receptor (IP3R) impairments and altered calcium homeostasis, with consequent inhibited mitochondrial fusion, altered mitochondrial trafficking, and increased mitophagy (30). Deficiency of WFS1 protein in MAMs and, more specifically an alteration of the interaction between WFS1 protein and Voltage-Dependent Anion Channel 1 (VDAC1), disrupts MAMs integrity, reduces Ca^2+^ transfer from the ER to mitochondria and impairs oxidative phosphorylation (31, 32).

Different mutations in the *WFS1* gene are associated with distinct clinical manifestations, with nonsense or frameshift variants generally resulting in more severe symptoms compared to missense or in-frame variants (33). Patient-derived induced pluripotent stem cells (iPSCs) represent a valuable *in vitro* model to investigate alternative splicing isoforms of WFS1 in pancreatic beta cells, as recently demonstrated by Chimienti et al. (33). In their study, the authors explored the molecular mechanisms underlying pathological alterations in a WS patient carrying compound heterozygous *WFS1* mutations: c.316-1G > A, affecting the acceptor splice site upstream of exon 4, and c.757A > T, introducing a premature termination codon (PTC) in exon 7. They demonstrated that partial retention of the functional Wolframin C-terminal domain preserves some endoplasmic reticulum (ER) stress responses. Furthermore, they reported that PTC-containing *WFS1* mRNAs undergo nonsense-mediated decay (NMD) in the beta cells, and showed that inflammatory cytokines exacerbate NMD, leading to increased beta-cell death.

Building upon these findings, a subsequent study delved deeper into the phenotypic characteristics of beta cells derived from the same patient. This research revealed that these cells exhibited an immature endocrine profile, defective pro-insulin processing, blunted glucose-stimulated Ca^2+^ oscillations, and altered autophagic flux. The study also highlighted the potential of patient-specific iPSC-derived beta cells as a platform for modeling disease pathology (34). A deeper understanding of genotype-phenotype correlations will advance our knowledge of Wolfram syndrome pathogenesis and potentially open new avenues for therapeutic intervention.

### 2.2 Characteristics of DM in WS1

DM in WS1 is considered similar to T1DM, as both are insulin-deficient and require insulin treatment to control hyperglycemia. Autoimmunity in DM related to WS1 is negative, except for rare cases of positivity reported in the literature (12). The onset of diabetes is usually early in life, and with a lower prevalence of diabetic ketoacidosis (14). Compared to T1DM, diabetes in WS1 has a longer phase of remission after onset, and patients are prone to hypoglycaemic events (35), likely because of an impairment of hypoglycemia awareness, as a result of the associated neurologic dysfunction. A measurable C-peptide secretion has been reported in some cases after more than 8 years from the diagnosis of diabetes related to WS1 (36). Glucose control is usually better and insulin doses are lower in DM associated WS1 than in T1DM. In the cohort of Cano et al. (14) mean HbA1c in WS1 patients was 7.72 ± 0.21 vs. 8.99 ± 0.25% in persons with T1DM, *P* = 0.002, while the mean insulin requirement was 0.71 ± 0.07 vs. 0.88 ± 0.04 UI * kg * day, respectively, *P* = 0.0325. Chronic diabetes complications are rarer in WS1 than in T1DM (14, 35). These data suggest that the progression of insulin deficiency may be slower in DM related to WS1 than in T1DM. In these patients, higher values of HbA1c are associated with a faster progression of neurodegenerative and endocrine symptoms (35), indicating that hyperglycaemia itself may play a role in ER distress. Glucose toxicity on different body tissues involves oxidative stress by reactive oxygen species (37, 38). This highlights the importance of good glucose control in WS1 patients. Table 1 summarizes characteristics of diabetes related to WS1 compared to T1DM.

**TABLE 1:** Characteristics of diabetes related to WS1 compared to Type 1 diabetes.

	WS1-related diabetes	T1DM
Age at onset	Earlier	Later
DKA at onset	Rare	Frequent
Autoantibodies for diabetes	Negative/rare	Present
HbA1c at onset	Low	High
Mean Hba1c during treatment	Low	High
Remission	Long	Short
Insulin requirement	Low	High
Diabetic retinopathy	Rare	More common
Diabetic nephropathy	Rare	More common

WS1, Wolfram syndrome; T1DM, type 1 diabetes; DKA, diabetic ketoacidosis.

### 2.3 Treatment of diabetes related to WS1

Presently, there are no therapies capable of interfering with the natural history of WS1, and treatment of WS1 relies on the treatment of associated conditions. Diabetes associated with WS1 requires insulin therapy, using a basal-bolus scheme or continuous subcutaneous insulin infusion (CSII) (11). Recently, other therapies have been tested to modulate WS1 pathogenic mechanisms and indirectly increase the synthesis of insulin and restore other damaged pathways.

As chemical chaperones have a role in ER distress, they have been tested as a possible regulator of ER distress in WS1. A chemical protein folding and trafficking chaperone, 4-phenyl butyric acid (PBA), has been studied in insulin-producing cells created from skin fibroblasts of patients with WS1 (39). The chaperone was effective in reducing the activity of UPR pathways and restoring insulin production. Two chaperones, 4-PBA and tauroursodeoxycholic acid (TUDCA), have already been approved by the Food and Drug Administration (FDA) and may have the potential to reduce misfolded WFS1 protein load (40, 41), but the need to use high concentrations may limit the use of these drugs in humans (41).

Another therapeutic target may be ER calcium stabilizers that can reduce the WFS1 calcium depletion and counteract the activation of the calpain protease pathway. Dantrolene sodium is an FDA-approved drug for malignant hyperthermia and muscle spasm (40). It inhibits calcium depletion through ryanodine receptors on the ER. It has been studied in a phase Ib/IIa open-label trial on 19 persons with WS1 (42). It was safe and well tolerated, but unfortunately not effective in improving beta-cell function, although there was a positive correlation between baseline beta-cell function and change in beta-cell function after 6 months. Furthermore, there were no improvements in visual acuity and neurological functions (42).

An interesting target could be p21 protein, a cyclin-dependent kinase inhibitor with anti-apoptotic effect, fundamental for cell survival after ER stress (41). Valproic acid is a drug approved for the treatment of epilepsy, bipolar disorder, and migraine. It is known to increase the expression of p21 in WS1 (43). A randomized double-blind, placebo-controlled trial on 63 persons with WS1 is currently ongoing to test valproate in WS1 (ClinicalTrials.gov identifier: NCT03717909). In case Valproate will be used in clinical practice in women with WS, it will be important to make patients aware of the teratogenic effects of this drug (44).

Glucagon-like peptide-1 receptor agonists (GLP-1 RA) may be a treatment for WS1 (45). These drugs are already prescribed to treat diabetes and obesity. GLP1-RA can prevent and reduce cell death due to ER stress *in vitro* and animal models (41). Toots et al. (46) demonstrated that a 6-month treatment with Liraglutide, started before the occurrence of the metabolic symptoms, prevented the development of glucose intolerance, ameliorated insulin and glucagon secretion, reduced ER stress and inflammation in beta cells of a rat model of WS1. Data on the use of Liraglutide in four pediatric patients with WS1 are reassuring in terms of safety, tolerability, and efficacy on C-peptide secretion and glucose metrics (47). A case report showed that treatment with liraglutide improved glycemic control and reduced daily insulin requirement by 20% in one patient with WS1 (48). A mechanistic study confirmed and expanded upon these findings, showing that liraglutide treatment not only effectively restored insulin secretion capacity but also ameliorated other critical cellular defects, including impaired autophagy and inflammation-driven apoptosis. These results underscore the potential of liraglutide as a multifaceted therapeutic strategy in managing the complex pathophysiological manifestations of WS1 (34).

Other promising approaches could be gene therapy and regenerative medicine (40). Adeno-associated viral (AAV) systems could be used to transfer wild-type WS1 and allow producing the native protein (40). Regenerative medicine should work to replace damaged tissues, especially beta cells and ganglion cells, as iPSCs have been generated from patients with WS1 and their relatives for scientific investigations (49), thus representing a valuable platform for replacement therapy (40).

Given the involvement of MAM dysfunction in WS1, therapeutic strategies are emerging that target ER–mitochondria communication and mitochondrial health. Pharmacological activation of the Sigma 1 receptor (S1R), an ER resident chaperone, restored calcium transfer from ER to mitochondria, improve mitochondrial respiration, and reduce pathological autophagy in WS1 models (50). In transgenic mouse and zebrafish models of WS1, the activation of S1R was obtained through the S1R agonist PRE-084 and allowed to reverse locomotor and cognitive deficits, highlighting a potential disease-modifying approach (50). Also liraglutide demonstrated a positive effect on calcium homeostasis and mitochondrial vitality (32).

## 3 Fertility in Wolfram syndrome

Infertility in WS1 is caused by gonadal dysfunction. Both hypogonadotropic and hypergonadotropic hypogonadism have been described (6, 7, 41, 51), and their prevalence varies from different studies, ranging from 7.1% to 50% (52–55). In the cohort of Salzano et al. (52), hypogonadotropic hypogonadism and hypergonadotropic hypogonadism were both reported in 7.1% of the patients. In a Turkish cohort (54), hypogonadism occurred in 33% of the patients, and similarly, in a Spanish cohort (53), hypogonadism was reported in 27.8% of patients with WS1. In an Italian cohort, primary hypogonadism reached a prevalence of 50% (55). Gonadal dysfunction is less frequent in females, who usually have primary amenorrhoea with low levels of gonadotropins, even if a case of hypergonadotropic hypogonadism has been reported in a 16-year-old female (56). Gonadal dysfunction in males has a variety of presentations, with fibrotic and atrophic testes, small penis, erectile dysfunction, and gynecomastia (41, 51–53). Pubertal and growth delay have also been reported due to hypopituitarism (55).

In WS1, there is an anterior pituitary hypofunction due to hypothalamic decreased function, causing secondary hypogonadism (11). The mechanism at the basis of primary hypogonadism is not clear. A hypothesis has been elaborated in studies in male mice knock out (KO) for the *Wolframin* gene (57). In *WFS1* KO mice, there is an alteration of sperm morphology, but no impairment in the motility of spermatozoa. Histology revealed that the seminiferous tubules in *WFS1* KO mice had no spermatogenic cells. The quantity of sperm was reduced due to a decrease in the number of spermatogonia and Sertoli cells. Leydig cells were not affected. Wolframin underexpression may impair spermatogenesis by increasing ER stress and spermatogenic cell apoptosis (55).

Despite these disorders in fertility, a case of male fertility (58) and various cases of pregnancies have been described (11).

## 4 Pregnancy in Wolfram syndrome

To date, there have been twenty reported cases of successful pregnancy in women with WS1 worldwide (6, 59–66). The available clinical characteristics of published pregnancies in WS1 females are summarized in Table 2.

**TABLE 2:** Characteristics of pregnancies in females affected by WS1 according to literature.

	Peden et al. (59)	Davidson et al. (60)	Davidson et al. (60)	Davidson et al. (60)	Wilson et al. (61)	Rugolo (62)	Kesavadev (63)	Toppings (64)	Zhang (65)	Zhang (65)
Year	1986	1993	1993	1993	1995	2002	2007	2018	2023	2023
Country	Canada	Ireland	Ireland	Ireland	Australia	Italy	India	Canada	China	China
Age at pregnancy	24	27	30	NA	25	26	28	39	25	31
Features of WFS	DM, OA, palpable bladder	DM, OA	DM, OA	DM, early hydroureter	DM, OA, DI, deafness, dilatation of the pelvicalyceal system	DM, deafness, OA	DM, OA, acute psychosis, DI, bilateral hydronephrosis and hydroureters	OA, cerebellar ataxia, deafness	DM, OA	DM, OA
Previous oligomenorrhea	No, with delayed puberty	No	No	Yes	NA	Yes	No	NA	NA	NA
Spontaneous pregnancy	Yes	Yes	Yes	Yes	Yes	Yes	Yes	Yes	Yes	Yes
Insulin therapy	MDI	MDI	MDI	NA	MDI	NA	Insulin pump	MDI	MDI	MDI
HbA1c at conception (mmol/mol)	NA	NA	NA	NA	100	NA	67	43	NA	49
Maternal outcomes	Preeclampsia, partial cranial DI, sensorineural deafness, acute urinary retention	No	Preeclampsia, DI, hearing loss, lower limb peripheral neuropathy, atonic bladder, hydroureteronephrosis	Leg oedema, DI, cerebral infarct driving to hemiplegia, high tone hearing loss, OA	No	DI	No	DM	Acute appendicitis	No
Fetal outcomes	No	No	No	Polyhydramnios	IUGR	Polyhydramnios	No	No	No	Abnormal fetal heart rate monitoring
Week at delivery	36	38	37	37	32	34	36	39	35	37
Cesarean section	Yes	Yes	Yes	Yes	Yes	Yes	Yes	Yes	Yes	Yes
Neonatal gender and weight	2,466 g, F	M	M	M	1,610 g, F	2,350 g, M	3,100 Kg, M	3,374 g, F	2,500 g	3,200 g, F

NA, not available; DM, diabetes mellitus; DI, diabetes insipidus; OA, optic atrophy; AUR, acute urinary retention; IUGR, intrauterine growth restriction.

The first report of a successful pregnancy in WS1 was described by Peden et al. (59). At the time of conception, the patient had already developed optic atrophy and diabetes mellitus. Despite delayed menarche, menses were regular, and she spontaneously conceived. During pregnancy, she developed partial cranial diabetes insipidus. Due to pre-eclampsia, she underwent a cesarean section at 36 gestational weeks, giving birth to a female infant of 2.460 grams, whom she breastfed for three weeks. During pregnancy, her hearing declined, and audiometry revealed severe sensorineural hearing loss. After childbirth and while not using desmopressin, she experienced recurrent episodes of urinary retention, temporarily requiring an indwelling catheter. Exams showed a dilated bladder, hydronephrosis, and hydroureter. WS spectrum disorder was confirmed.

In 1993, Davidson et al. (60) described two siblings with WS1. The first sibling was diagnosed with diabetes at the age of 13 and optic atrophy at 19. She experienced menarche at 16 and had regular menstrual cycles. At age 27, she became pregnant and delivered a healthy boy via cesarean section. At 30, she became pregnant again, and her pregnancy was complicated by pre-eclampsia. After delivery, she was diagnosed with diabetes insipidus and hearing loss, leading to the diagnosis of WS. The younger sister was diagnosed with diabetes at the age of 5. She experienced menarche at 17 with irregular menstrual cycles. At 21, she became pregnant. Her pregnancy was complicated by polyhydramnios and oedema of the lower extremities, and she gave birth to a baby boy through cesarean section. Following this birth, she developed severe diabetes insipidus, hypovolemia, and a cerebral infarction. She was subsequently diagnosed with hearing loss and optic atrophy.

In 1995, Wilson and Moore (61) reported another case of successful pregnancy in a patient with WS1. Her Hb1Ac at conception was elevated, but improved during pregnancy. At 32 weeks of gestation, ultrasound examination revealed significant fetal growth restriction and impaired diastolic blood flow in the umbilical cord. Dexamethasone therapy was administered for fetal pulmonary maturation, and she delivered a female infant, weighing 1,610 g, by cesarean section at 32 gestational weeks.

In 1995, Barrett et al. (6) performed a nationwide cross-sectional case-finding study in the United Kingdom. Many female patients with WS1 exhibited menstrual irregularities and delayed menarche. Five of them had successful pregnancies, resulting in the birth of nine unaffected children. No further clinical information about their pregnancies is available.

In 2002, Rugolo et al. (62) described the pregnancy of a 26-year-old female patient with WS1. The patient was known for diabetes mellitus, optic atrophy, and deafness. During pregnancy was diagnosed with diabetes insipidus, so a diagnosis of WS1 was made. Unfortunately, at 33 gestational weeks, ultrasound examination revealed polyhydramnios, elevated fibrinogen levels, alterations in the electrophoretogram, and worsening glycemic control, prompting the decision to perform a cesarean section at 34 weeks of gestation. This resulted in the birth of a healthy male infant weighing 2,350 g.

An Indian patient previously diagnosed with WS1 was followed before and during her pregnancy in 2006 (63). During prenatal care, she was initially started on a basal-bolus regimen (glargine and three times-daily premeal insulin aspart), later changed to a subcutaneous insulin pump in association with frequent capillary blood glucose monitoring, maintaining HbA1c between 5.2% and 5.9%. She became pregnant in December 2006, and HbA1c remained below 6% throughout her pregnancy. She was admitted to the hospital in the final month of her pregnancy. A cesarean section was performed, delivering a healthy male baby weighing 3.100 grams. She had an uneventful postpartum period.

In 2018, Toppings et al. (64) published the case of a 39-year-old woman who was diagnosed with WS1 after being evaluated following her first pregnancy complicated by gestational diabetes (GDM). She was known to have bilateral optic atrophy and sensorineural deafness from the ages of 10 and 25 years, respectively. She also presented neurological manifestations compatible with cerebellar ataxia. She was diagnosed with GDM at 16 weeks of gestation, with age being her only risk factor for GDM. She was treated with insulin, up to a maximum of 25 units per day. She delivered a healthy female neonate weighing 3,374 g at 39 weeks of gestation by cesarean section due to transverse presentation. Three months postpartum, the patient met the criteria for diagnosis of diabetes mellitus based on a 75 g oral glucose tolerance test (OGTT). Antiglutamic acid decarboxylase and anti-islet cell antibodies were negative. Genetic testing revealed two mutations pathognomonic for WS1.

The most recent report was the one about two subsequent pregnancies in a female known for WS1 since the age of 24 (65). The patient had three induced abortions for personal reasons. At 27 years of age, she delivered her first son by emergency cesarean section due to acute appendicitis at 35 weeks of gestation. Her second pregnancy started with an HbA1c of 6.6%; she was treated with intensive insulin therapy and reached and maintained HbA1c levels of 5.6%–6% throughout pregnancy. She was hospitalized in the third trimester due to an abnormality in the fetal heart rate monitoring. A cesarean section delivery was performed at 37 weeks of gestation because of breech presentation. There were no adverse maternal or fetal outcomes.

## 5 Case report

A 44-year-old woman with Wolfram syndrome type 1 (WS1) spontaneously became pregnant. Diabetes mellitus was diagnosed at the age of 11 and was initially managed with nutritional therapy. However, glycaemic control gradually worsened, requiring insulin therapy. At age 35, due to frequent nocturnal hypoglycaemic episodes, she was started on an insulin pump with continuous glucose monitoring (CGM). Her insulin pump evolved from a system with low-glucose suspend to predictive low-glucose suspend, and eventually to an advanced hybrid closed-loop (AHCL) system (MiniMed 780G). At age 32, she developed urinary symptoms including urgency, frequency, and enuresis. Urodynamic evaluation led to a diagnosis of detrusor overactivity, and anticholinergic treatment was initiated. That same year, brain magnetic resonance imaging revealed multisystemic brain atrophy. Progressive ophthalmologic issues also emerged, including high myopia with nystagmus, elevated intraocular pressure, and optic atrophy. At age 39, the diagnosis of WS1 was genetically confirmed. Two heterozygous pathogenic variants in exon 8 of the *WFS1* gene were identified: a three-nucleotide deletion (c.1060_1062del), resulting in the loss of one amino acid (p.Phe354del), and a missense mutation (c.2534T > G), leading to an amino acid substitution (p.Ile845Ser).

At 41 years of age, the patient had regular menstrual cycles every 34–38 days and began planning for pregnancy at the multidisciplinary Diabetes and Pregnancy outpatient clinic of our hospital. Glycemic control improved following adjustments to her therapy and participation in educational sessions, with HbA1c reaching near-target levels at 6.8% (51 mmol/mol). Anticholinergic therapy, which is contraindicated in pregnancy, was discontinued, and folic acid supplementation at a dose of 5 mg daily was initiated. Her partner underwent genetic counseling to assess the risk of WS1 transmission to their offspring.

**FIGURE 1:**
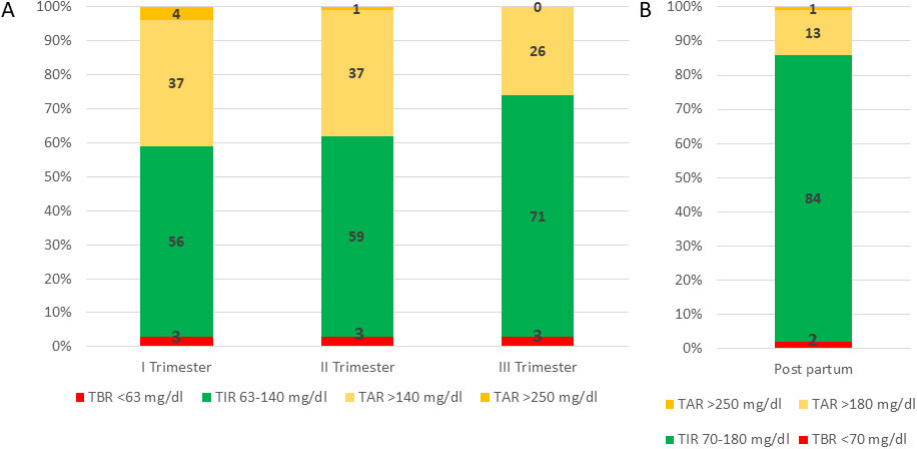
Time in range (TIR), time below range (TBR) and time above range (TAR) during pregnancy (**Panel A**) and in the post-partum period (**Panel B**).

Despite initial efforts, spontaneous conception did not occur, and the patient underwent assisted reproductive therapy three years later, which was unsuccessful, but at the age of 44, she conceived spontaneously. Her first antenatal visit was at 8 weeks of gestation. Preconception HbA1c was 7.2% (55 mmol/mol), and her preconception weight was 46 kg (height 1.56 m; Body Mass Index [BMI]: 18.6). The patient was informed about the off-label use of the MiniMed 780G advanced hybrid closed-loop (AHCL) system during pregnancy and agreed to continue using it with the SmartGuard algorithm. The duration of insulin on board was reduced to 2 h, and the glycemic target was set at 100 mg/dL. Despite educational support, adherence to continuous glucose monitoring (CGM) sensor use was suboptimal. She continued to use carbohydrate counting, and insulin-to-carbohydrate (IC) ratios were adjusted throughout pregnancy in response to rising insulin requirements and increasing insulin resistance (Table 3). With support from a dietitian, food diaries were regularly reviewed; however, adherence to nutritional recommendations remained suboptimal. In each trimester, automated correction boluses delivered by the algorithm accounted for more than 33% of the total daily insulin dose (TDD). The patient was advised to administer manual boluses when automated corrections were insufficient. Pregnancy-specific time in range (psTIR) was below target during the first and second trimesters but reached the desired level in the third trimester when SmartGuard usage exceeded 90% of the time (Figure 1). Her weight increased from 49.8 kg at the end of the first trimester to 53.6 kg at the end of the second trimester, reaching 68 kg by the end of pregnancy.

**TABLE 3:** Insulin pump settings and glucose indexes during pregnancy and post-partum.

	First trimester	Second trimester	Third trimester	Post-partum
Use of SmartGuard, %	83	89	91	73
Avg ± SD sensor glucose, mg/dl	137 ± 54	130 ± 44	117 ± 37	135 ± 43
Avg ± SD capillary glucose, mg/dl	139 ± 53	128 ± 47	117 ± 43	153 ± 59
CV, %	39.6	34.2	31.5	31.9
Avg ± SD daily CHO, gr	253 ± 43	237 ± 34	238 ± 73	221 ± 50
GMI, % (mmol/mol)	6.6 (48.6)	6.4 (46.4)	6.1 (43.2)	6.5 (47.5)
HbA1c, % (mmol/mol)	7 (53)	46 (6.4)	42 (6)	40 (5.8)
TDD, units	25.6	24.6	28.5	23.3
Insulin for boluses, units (%)	15.5 (61)	15.9 (65)	19.1 (67)	13.5 (58)
Insulin for basal, units (%)	3.1 (20)	3.2 (20)	2.2 (12)	2.3 (17)
Insulin for correction boluses, units (%)	10.1 (39)	8.7 (35)	9.4 (33)	9.8 (42)
I/C ratio at breakfast	25	16	20	20
I/C ratio at lunch	11	12	20	18
I/C ratio at dinner	25	22	30	26

Avg, average; SD, standard deviation; CHO, carbohydrate; CV, coefficient of variation; GMI, glucose management index; HbA1c, glycated hemoglobin; TDD, total daily dose; I/C, insulin/carbohydrate.

From the first antenatal visit, low-dose aspirin 100 mg daily was prescribed for the prevention of preeclampsia. Doxylamine combined with pyridoxine was used during the first trimester to manage nausea and vomiting. At 16 weeks of gestation, the patient was treated with antibiotics for a urinary tract infection. At 22 weeks, she developed a cough and fever, which were managed with paracetamol and aerosol therapy, including topical corticosteroids and mucolytics. Starting at 30 weeks, she began daily fetal movement counting, and from 32 weeks, routine fetal monitoring with non-stress tests was initiated. At 37 weeks and 2 days, elevated blood pressure (ranging from 155–186/82–98 mmHg) was detected during a non-stress test. Laboratory evaluation revealed elevated liver enzymes—alanine transaminase (ALT) at 85 U/L (normal: 6–41) and aspartate transaminase (AST) at 98 U/L (normal: 5–33)—a sFlt1/PlGF ratio of 153, and proteinuria of 0.686 g/24 h. A diagnosis of preeclampsia was made, and antihypertensive treatment with a calcium channel blocker was initiated. An elective cesarean section was performed the following day, at 37 weeks and 3 days of gestation. The patient delivered a healthy female neonate weighing 3,275 grams, with Apgar scores of 8 at 1 min and 10 at 5 min. PH of the umbilical artery was 7.129 U, base excess was −2.5 mmol/L. The newborn experienced transient respiratory distress, which resolved within 3 min of assisted ventilation. Hypoglycemia occurred at 2 h postpartum and was successfully treated with a protein hydrolysate formula. Two days after delivery, the patient continued to experience hypertension, leg oedema, and hypoalbuminemia. Nephrology consultation led to the addition of furosemide and an ACE inhibitor, alongside the existing calcium antagonist therapy. All antihypertensive medications were discontinued within one month. Soon after delivery, antispasmodic therapy for bladder dysfunction was resumed, following evaluation by a urologist and pelvic floor specialists. The patient opted for formula feeding and did not breastfeed.

At the 6-week postpartum follow-up, she maintained good glycemic control (Figure 1 and Table 3).

## 6 Discussion

WS1 is a progressive neurodegenerative disease affecting different organs. The diagnosis is often delayed until several conditions associated with this specific mutation are evident and a genetic test is performed. Current management relies on symptomatic treatment and hormone replacement therapy, depending on the clinical picture. Daily life for patients with this rare disease is often challenging due to insulin-treated diabetes, visual and hearing impairments, the need for multiple medications, and frequent outpatient visits and examinations.

Despite the presence of gonadal dysfunction, spontaneous conception in patients with WS is possible; however, these pregnancies are considered high-risk. Pre-gestational diabetes confers a higher probability of developing maternal and fetal/neonatal adverse outcomes, such as spontaneous abortion, malformations, macrosomia, pre-eclampsia, and requires strict glucose control (67). Furthermore, during pregnancy, females with WS1 are also required to manage WS1-associated conditions, such as urinary tract abnormalities.

During a high-risk pregnancy, a multidisciplinary approach is highly recommended. Our patient was followed by a team of specialists, including an endocrinologist and a dietitian expert in diabetes pregnancy, and a gynecologist. After delivery, the patient needed an evaluation from a nephrologist and a urologist. Therefore, pregnancy in WS1 should be followed at a tertiary care center with expertise in rare diseases and high-risk pregnancy.

Our patient experienced pre-eclampsia at the end of pregnancy. This also occurred in the other two cases reported in the literature (Table 2). Almost all of the other cases reported in literature experienced pregnancy complications, i.e., negative fetal outcomes as polyhydramnios and fetal growth restriction, and almost all deliveries required cesarean section. Immunohistochemical expression of Wolframin in human placenta throughout pregnancy in normal women and pregnant women with diabetes has been studied by Lucariello et al. (68). There is a modulation of human placental Wolframin during pregnancy, which is strongly expressed in the first trimester and moderately expressed in the third trimester. In women with diabetes, the decrease in Wolframin placental expression observed in the third trimester of gestation was greater than in healthy women. Wolframin may be required to sustain normal rates of cytotrophoblast cell proliferation. Histological evaluation of the placenta of our patient revealed placental parenchyma with maturity of chorionic villi, consistent with the third trimester of gestation, with some areas of increased inter-villous spaces, discrete and diffuse Chorangiosis, and modest inter-villous fibrinosis. The umbilical cord presented a modest stromal edema.

Few cases of successful pregnancies are reported in the literature, although the entity of spontaneous conception in WS is not clear. It may be possible that the prevalence of spontaneous pregnancy in WS1 is higher than expected. As for other conditions (i.e., Prader–Willi Syndrome, transplant, etc), an international registry collecting data on the pregnancies in women with WS1 would inform patients’ decisions and healthcare professionals’ counseling. WS1 has, unfortunately, a poor prognosis with a limited life expectancy (6, 11), although in the last decades, improvements in diagnosis and treatment may have contributed to extending life and improving the quality of life of all patients with WS1, including women with WS1 of reproductive age. Assisted reproductive technology is now widespread, and this approach may help females with WS1 and fertility issues conceive, increasing pregnancy rates in this rare disease. Moreover, technology in the management of diabetes, such as the integration between insulin pumps and CGM, which has led to automated insulin delivery system (AID), has been making optimal glucose control reachable by a larger number of patients than in the past (69), when only traditional multiple daily injection (MDI) therapy or stand-alone insulin pump (69) were available. These results have been reported independently of age, gender, duration of diabetes, former therapy, or start of glucose control (70). Better glucose control is associated with slower progression of neurodegeneration in WS (13, 35). Diabetes technology has also transformed the management of diabetes during pregnancy. To date, only the CamAPS FX system has strong evidence for the use in pregnancy (71), although trials on the use of other AHCL systems in pregnancy have been recently published. In the AIDAPT trial (71), 124 participants were randomly assigned to AID therapy with the CamAPS algorithm or standard care. Women in the AID group reached higher pregnancy-specific TIR without an increase in adverse events (71). In the CRISTAL trial, 95 pregnant females with diabetes were randomly assigned to AHCL therapy using Minimed 780G with SmartGuard algorithm or standard insulin therapy (72). The use of AHCL improved overnight time in the target range, reduced time below range, and improved treatment satisfaction (71). At the time of our patient’s pregnancy, evidence on AHCL use in pregnancy was not available, and the patient, adequately informed, decided to maintain her usual therapy with Minimed 780G.

Planning pregnancy in cases of pre-gestational diabetes is paramount to optimize glucose control, start folic acid supplementation, screen for complications, and change or interrupt drugs contraindicated in pregnancy (73). Pre-pregnancy care in WS1 is even more important because of the additional burden of neurological and ophthalmological conditions associated with WS1. Furthermore, women with WS1 often take oral drugs for treatment of WS1-associated conditions that are not recommended for use in pregnancy, and, therefore, it is important to find alternatives, when possible, before conception. Genetic consultation for the partner is very important and should be performed before conception. No genetic screening of the newborn is usually recommended, unless both parents are carriers of the mutation. The risk of a *de novo* mutation is less than 1%. However, in our patient two heterozygous variants were identified in exon 8 of the *WFS1* gene: a three-nucleotide in-frame deletion (c.1060_1062del), leading to the loss of a phenylalanine at position 354 (p.Phe354del), and a missense mutation (c.2534T > G), resulting in the substitution of isoleucine with serine at position 845 (p.Ile845Ser). The p.Phe354del variant removes a conserved hydrophobic residue, potentially altering the local protein structure and affecting the function of Wolframin in ER homeostasis, particularly if the deleted residue lies in a domain important for calcium regulation or protein folding. The p.Ile845Ser variant replaces a non-polar amino acid with a polar one within the C-terminal ER-luminal domain of Wolframin, a region known to mediate cellular responses to ER stress; this substitution may impair Wolframin’s ability to maintain ER function and cell survival. The p.Phe354del alteration, previously described by Cano et al. (74), disrupts the first transmembrane domain of Wolframin and has been reported in a patient presenting neurogenic bladder, ataxia, dysautonomia, and psychiatric disturbances. The p.Ile845Ser change occurs at the same residue where the p.Ile845Asn variant (c.2534T > A) was identified in homozygosity in a patient with hydronephrosis, bilateral neuropathic pain, early-onset diabetes mellitus, cataract, and optic atrophy (75). Given the distinct biochemical properties of serine versus asparagine, we anticipate that p.Ile845Ser may confer functionally unique effects on Wolframin stability and cellular homeostasis, particularly influencing C-terminal–mediated processes implicated in the progressive ocular and renal phenotypes observed. Although *WFS1*-related Wolfram syndrome is classically recessive, several heterozygous *WFS1* mutations—particularly in-frame deletions and C-terminal missense variants—have been associated with dominant phenotypes such as non-syndromic sensorineural hearing loss, psychiatric symptoms, or adult-onset diabetes. Therefore, even if transmitted independently, each of these variants could potentially manifest in the offspring as an isolated, mild, or late-onset clinical feature, including sensorineural hearing loss, glucose intolerance or diabetes, or neuropsychiatric traits, due to dominant-negative effects or partial loss of function.

In conclusion, diabetes mellitus related to WS1 is a form of non-autoimmune diabetes characterized by a progressive loss of pancreatic beta-cell mass and function, driven by ER stress and other intertwined processes, including impaired autophagy, dysregulated calcium homeostasis, mitochondrial dysfunction, and chronic inflammation. The clinical management of patients with WS1 is challenging, both because of phenotypic heterogeneity and the presence of various comorbidities. Optimal glucose control is essential to slow the progression of neurodegenerative complications and improve quality of life. Spontaneous pregnancy in these women is possible, although rare. With careful multidisciplinary management, it is possible to achieve positive outcomes. These considerations underline the importance of early diagnosis, specialist management, and continuous research into new therapeutic strategies for a disease still without a cure.

## Data Availability

The raw data supporting the conclusions of this article will be made available by the authors, without undue reservation.

## References

[1] Wolfram D Wagener H. Diabetes mellitus and simple optic atrophy among siblings: report of four cases. *Mayo Clin Proc.* (1938) 13:715–8.

[2] Pilley S Thompson H. Familial syndrome of diabetes insipidus, diabetes mellitus, optic atrophy, and deafness (didmoad) in childhood. *Br J Ophthalmol.* (1976) 60:294–8. 10.1136/bjo.60.4.294 1276119 PMC1017495

[3] Kumar S. Wolfram syndrome: important implications for pediatricians and pediatric endocrinologists. *Pediatr Diabetes.* (2010) 11:28–37. 10.1111/j.1399-5448.2009.00518.x 20015125

[4] Fraser F Gunn T. Diabetes mellitus, diabetes insipidus, and optic atrophy. An autosomal recessive syndrome? *J Med Genet.* (1977) 14:190–3. 10.1136/jmg.14.3.190 881709 PMC1013555

[5] Kinsley B Swift M Dumont R Swift R. Morbidity and mortality in the Wolfram syndrome. *Diabetes Care.* (1995) 18:1566–70. 10.2337/diacare.18.12.1566 8722052

[6] Barrett T Bundey S Macleod A. Neurodegeneration and diabetes: UK nationwide study of Wolfram (DIDMOAD) syndrome. *Lancet.* (1995) 346:1458–63. 10.1016/s0140-6736(95)92473-6 7490992

[7] Rigoli L Aloi C Salina A Di Bella C Salzano G Caruso R Wolfram syndrome 1 in the Italian population: genotype-phenotype correlations. *Pediatr Res.* (2020) 87:456–62. 10.1038/s41390-019-0487-4 31266054

[8] Lombardo F Salzano G Di Bella C Aversa T Pugliatti F Cara S Phenotypical and genotypical expression of Wolfram syndrome in 12 patients from a Sicilian district where this syndrome might not be so infrequent as generally expected. *J Endocrinol Invest.* (2014) 37:195–202. 10.1007/s40618-013-0039-4 24497219

[9] Takei D Ishihara H Yamaguchi S Yamada T Tamura A Katagiri H WFS1 protein modulates the free Ca(2+) concentration in the endoplasmic reticulum. *FEBS Lett.* (2006) 580:5635–40. 10.1016/j.febslet.2006.09.007 16989814

[10] Amr S Heisey C Zhang M Xia X Shows K Ajlouni K A homozygous mutation in a novel zinc-finger protein, ERIS, is responsible for Wolfram syndrome 2. *Am J Hum Genet.* (2007) 81:673–83. 10.1086/520961 17846994 PMC2227919

[11] Frontino G Delvecchio M Prudente S Sordi V Barboni P Di Giamberardino A SID/SIEDP expert consensus on optimizing clinical strategies for early detection and management of wolfram syndrome. *J Endocrinol Invest.* (2025) 48:507–25. 10.1007/s40618-024-02495-z 39527371 PMC11876246

[12] Nakamura A Shimizu C Nagai S Taniguchi S Umetsu M Atsumi T A novel mutation of WFS1 gene in a Japanese man of Wolfram syndrome with positive diabetes-related antibodies. *Diabetes Res Clin Pract.* (2006) 73:215–7. 10.1016/j.diabres.2005.12.007 16442662

[13] Iafusco D Zanfardino A Piscopo A Curto S Troncone A Chianese A Metabolic treatment of Wolfram syndrome. *Int J Environ Res Public Health.* (2022) 19:2755. 10.3390/ijerph19052755 35270448 PMC8910219

[14] Cano A Molines L Valéro R Simonin G Paquis-Flucklinger V Vialettes B Microvascular diabetes complications in Wolfram syndrome (diabetes insipidus, diabetes mellitus, optic atrophy, and deafness [DIDMOAD]): an age- and duration-matched comparison with common type 1 diabetes. *Diabetes Care.* (2007) 30:2327–30. 10.2337/dc07-0380 17536072

[15] Pallotta M Tascini G Crispoldi R Orabona C Mondanelli G Grohmann U Wolfram syndrome, a rare neurodegenerative disease: from pathogenesis to future treatment perspectives. *J Transl Med.* (2019) 17:238. 10.1186/s12967-019-1993-1 31337416 PMC6651977

[16] Zatyka M Da Silva Xavier G Bellomo EA Leadbeater W Astuti D Smith J Sarco(endo)plasmic reticulum ATPase is a molecular partner of Wolfram syndrome 1 protein, which negatively regulates its expression. *Hum Mol Genet.* (2015) 24:814–27. 10.1093/hmg/ddu499 25274773 PMC4291252

[17] Angebault C Fauconnier J Patergnani S Rieusset J Danese A Affortit C ER-mitochondria cross-talk is regulated by the Ca2+ sensor NCS1 and is impaired in Wolfram syndrome. *Sci Signal.* (2018) 11:eaaq1380. 10.1126/scisignal.aaq1380 30352948

[18] Fonseca S Fukuma M Lipson K Nguyen L Allen J Oka Y WFS1 is a novel component of the unfolded protein response and maintains homeostasis of the endoplasmic reticulum in pancreatic beta-cells. *J Biol Chem.* (2005) 280:39609–15. 10.1074/jbc.M507426200 16195229

[19] Wang L Liu N Zhang X Song E Wang T Xu Z WFS1 functions in ER export of vesicular cargo proteins in pancreatic beta-cells. *Nat. Commun.* (2021) 12:6996. 10.1038/s41467-021-27344-y 34848728 PMC8632972

[20] Harding H Ron D. Endoplasmic reticulum stress and the development of diabetes: a review. *Diabetes.* (2002) 51:S455–61. 10.2337/diabetes.51.2007.s455 12475790

[21] Lin J Li H Yasumura D Cohen H Zhang C Panning B IRE1 signaling affects cell fate during the unfolded protein response. *Science.* (2007) 318:944–9. 10.1126/science.1146361 17991856 PMC3670588

[22] Han D Lerner A Vande Walle L Upton J Xu W Hagen A IRE1alpha kinase activation modes control alternate endoribonuclease outputs to determine divergent cell fates. *Cell.* (2009) 138:562–75. 10.1016/j.cell.2009.07.017 19665977 PMC2762408

[23] Hara T Mahadevan J Kanekura K Hara M Lu S Urano F. Calcium efflux from the endoplasmic reticulum leads to β-cell death. *Endocrinology.* (2014) 155:758–68. 10.1210/en.2013-1519 24424032 PMC3929724

[24] Abreu D Asada R Revilla J Lavagnino Z Kries K Piston D Wolfram syndrome 1 gene regulates pathways maintaining beta-cell health and survival. *Lab Invest.* (2020) 100:849–62. 10.1038/s41374-020-0408-5 32060407 PMC7286786

[25] Amo-Shiinoki K Tanabe K Nishimura W Hatanaka M Kondo M Kagawa S β cell dedifferentiation, the underlying mechanism of diabetes in Wolfram syndrome. *Sci Transl Med.* (2025) 17:ead2332. 10.1126/scitranslmed.adp2332 39970233

[26] Liu T Zhang L Joo D Sun SC. NF-κB signaling in inflammation. *Signal Transduct Target Ther.* (2017) 2:17023. 10.1038/sigtrans.2017.23 29158945 PMC5661633

[27] Garg A Kaczmarek A Krysko O Vandenabeele P Krysko D Agostinis PER. stress-induced inflammation: does it aid or impede disease progression? *Trends Mol Med.* (2012) 18:589–98. 10.1016/j.molmed.2012.06.010 22883813

[28] Yong J Johnson J Arvan P Han J Kaufman R. Therapeutic opportunities for pancreatic β-cell ER stress in diabetes mellitus. *Nat Rev Endocrinol.* (2021) 17:455–67. 10.1038/s41574-021-00510-4 34163039 PMC8765009

[29] Morikawa S Blacher L Onwumere C Urano F. Loss of function of WFS1 causes ER stress-mediated inflammation in pancreatic beta-cells. *Front Endocrinol.* (2022) 13:849204. 10.3389/fendo.2022.849204 35399956 PMC8990750

[30] Cagalinec M Liiv M Hodurova Z Hickey M Vaarmann A Mandel M Role of mitochondrial dynamics in neuronal development: mechanism for Wolfram syndrome. *PLoS Biol.* (2016) 14:e1002511. 10.1371/journal.pbio.1002511 27434582 PMC4951053

[31] Zatyka M Rosenstock T Sun C Palhegyi A Hughes G Lara-Reyna S Depletion of WFS1 compromises mitochondrial function in hiPSC-derived neuronal models of Wolfram syndrome. *Stem Cell Rep.* (2023) 18:1090–106. 10.1016/j.stemcr.2023.04.002 37163979 PMC10202695

[32] Liiv M Vaarmann A Safiulina D Choubey V Gupta R Kuum M ER calcium depletion as a key driver for impaired ER-to-mitochondria calcium transfer and mitochondrial dysfunction in Wolfram syndrome. *Nat Commun.* (2024) 15:6143. 10.1038/s41467-024-50502-x 39034309 PMC11271478

[33] Chimienti R Torchio S Siracusano G Zamarian V Monaco L Lombardo M A WFS1 variant disrupting acceptor splice site uncovers the impact of alternative splicing on beta cell apoptosis in a patient with Wolfram syndrome. *Diabetologia.* (2025) 68:128–51. 10.1007/s00125-024-06307-0 39520565 PMC11663190

[34] Torchio S Siracusano G Cuozzo F Zamarian V Pellegrini S Manenti F Liraglutide treatment reverses unconventional cellular defects in induced pluripotent stem cell-derived β-Cells harboring a partially functional WFS1 variant. *Diabetes.* (2025) 74:1273–88. 10.2337/db24-0720 40202504 PMC12185971

[35] Rohayem J Ehlers C Wiedemann B Holl R Oexle K Kordonouri O Diabetes and neurodegeneration in Wolfram syndrome: a multicenter study of phenotype and genotype. *Diabetes Care.* (2011) 34:1503–10. 10.2337/dc10-1937 21602428 PMC3120194

[36] Fishman L Ehrlich R. Wolfram syndrome: report of four new cases and a review of literature. *Diabetes Care.* (1986) 9:405–8. 10.2337/diacare.9.4.405 3461931

[37] Robertson R Harmon J. Diabetes, glucose toxicity, and oxidative stress: a case of double jeopardy for the pancreatic islet beta cell. *Free Radic Biol Med.* (2006) 41:177–84. 10.1016/j.freeradbiomed.2005.04.030 16814095

[38] Johnson J Luciani D. Mechanisms of pancreatic beta-cell apoptosis in diabetes and its therapies. *Adv Exp Med Biol.* (2010) 654:447–62. 10.1007/978-90-481-3271-3_19 20217509

[39] Shang H Foo K Martinez H Watanabe K Zimmer M β-cell dysfunction due to increased ER stress in a stem cell model of Wolfram syndrome. *Diabetes.* (2014) 63:923–33. 10.2337/db13-0717 24227685 PMC3931392

[40] Abreu D Urano F. Current landscape of treatments for Wolfram syndrome. *Trends Pharmacol Sci.* (2019) 40:711–4. 10.1016/j.tips.2019.07.011 31420094 PMC7547529

[41] Rigoli L Caruso V Salzano G Lombardo F. Wolfram syndrome 1: from genetics to therapy. *Int J Environ Res Public Health.* (2022) 19:3225. 10.3390/ijerph19063225 35328914 PMC8949990

[42] Abreu D Stone S Pearson T Bucelli R Simpson A Hurst S A phase Ib/IIa clinical trial of dantrolene sodium in patients with Wolfram syndrome. *JCI Insight.* (2021) 6:e145188. 10.1172/jci.insight.145188 34185708 PMC8410026

[43] Gharanei S Zatyka M Astuti D Fenton J Sik A Nagy Z Vacuolar-type H+-ATPase V1A subunit is a molecular partner of Wolfram syndrome 1 (WFS1) protein, which regulates its expression and stability. *Hum Mol Genet.* (2013) 22:203–17. 10.1093/hmg/dds400 23035048

[44] Bromley R Adab N Bluett-Duncan M Clayton-Smith J Christensen J Edwards K Monotherapy treatment of epilepsy in pregnancy: congenital malformation outcomes in the child. *Cochrane Database Syst Rev.* (2023) 8:CD010224. 10.1002/14651858.CD010224.pub3 37647086 PMC10463554

[45] Panfili E Frontino G Pallotta MT. GLP-1 receptor agonists as promising disease-modifying agents in WFS1 spectrum disorder. *Front Clin Diabetes Healthc.* (2023) 4:1171091. 10.3389/fcdhc.2023.1171091 37333802 PMC10275359

[46] Toots M Seppa K Jagomäe T Koppel T Pallase M Heinla I Preventive treatment with liraglutide protects against development of glucose intolerance in a rat model of Wolfram syndrome. *Sci Rep.* (2018) 8:10183. 10.1038/s41598-018-28314-z 29976929 PMC6033861

[47] Frontino G Raouf T Canarutto D Tirelli E Di Tonno R Rigamonti A Case report: off-label liraglutide use in children with wolfram syndrome type 1: extensive characterization of four patients. *Front Pediatr.* (2021) 9:755365. 10.3389/fped.2021.755365 34970515 PMC8712700

[48] Kondo M Tanabe K Amo-Shiinoki K Hatanaka M Morii T Takahashi H Activation of GLP-1 receptor signalling alleviates cellular stresses and improves beta cell function in a mouse model of Wolfram syndrome. *Diabetologia.* (2018) 61:2189–201. 10.1007/s00125-018-4679-y 30054673

[49] Lu S Kanekura K Hara T Mahadevan J Spears L Oslowski C A calcium-dependent protease as a potential therapeutic target for Wolfram syndrome. *Proc Natl Acad Sci U S A.* (2014) 111:E5292–301. 10.1073/pnas.1421055111 25422446 PMC4267371

[50] Crouzier L Danese A Yasui Y Richard E Liévens J Patergnani S Activation of the sigma-1 receptor chaperone alleviates symptoms of Wolfram syndrome in preclinical models. *Sci Transl Med.* (2022) 14:eabh3763. 10.1126/scitranslmed.abh3763 35138910 PMC9516885

[51] Homan M MacKay B. Primary hypogonadism in two siblings with Wolfram syndrome. *Diabetes Care.* (1987) 10:664–5. 10.2337/diacare.10.5.664 3677989

[52] Salzano G Rigoli L Valenzise M Chimenz R Passanisi S Lombardo F. Clinical peculiarities in a cohort of patients with Wolfram syndrome 1. *Int J Environ Res Public Health.* (2022) 19:520. 10.3390/ijerph19010520 35010780 PMC8744633

[53] Bueno G Ruiz-Castañeda D Martínez J Muñoz M Alascio P. Natural history and clinical characteristics of 50 patients with Wolfram syndrome. *Endocrine.* (2018) 61:440–6. 10.1007/s12020-018-1608-2 29728875

[54] Simsek E Simsek T Tekgül S Hosal S Seyrantepe V Aktan G. Wolfram (DIDMOAD) syndrome: a multidisciplinary clinical study in nine Turkish patients and review of the literature. *Acta Paediatr.* (2003) 92:55–61. 10.1111/j.1651-2227.2003.tb00469.x 12650300

[55] Frontino G Di Tonno R Stancampiano M Arrigoni F Rigamonti A Morotti E Paediatric Wolfram syndrome Type 1: should gonadal dysfunction be part of the diagnostic criteria? *Front Endocrinol.* (2023) 14:1155644. 10.3389/fendo.2023.1155644 37383390 PMC10294676

[56] Jodoin A Marchand M Beltrand J. Wolfram syndrome in a young woman with associated hypergonadotropic hypogonadism - A case report. *J Pediatr Endocrinol Metab.* (2022) 35:1552–5. 10.1515/jpem-2022-0268 36100371

[57] Noormets K Kõks S Kavak A Arend A Aunapuu M Keldrimaa A Male mice with deleted Wolframin (Wfs1) gene have reduced fertility. *Reprod Biol Endocrinol.* (2009) 7:82. 10.1186/1477-7827-7-82 19664290 PMC2734842

[58] Haghighi A Haghighi A Setoodeh A Saleh-Gohari N Astuti D Barrett T. Identification of homozygous WFS1 mutations (p.Asp211Asn, p.Gln486*) causing severe Wolfram syndrome and first report of male fertility. *Eur J Hum Genet.* (2013) 21:347–51. 10.1038/ejhg.2012.154 22781099 PMC3573194

[59] Peden N Gay J Jung R Kuwayti K. Wolfram (DIDMOAD) syndrome: a complex long-term problem in management. *Q J Med.* (1986) 58:167–80. 3086928

[60] Davidson I McNicholl J O’Donnell J. Successful pregnancy in two sisters with Wolfram syndrome. *Ir Med J.* (1993) 86:8444596. 8444596

[61] Wilson J Moore G. Successful pregnancy in the DIDMOAD syndrome (diabetes insipidus, diabetes mellitus, optic atrophy, deafness). *Aust N Z J Obstet Gynaecol.* (1995) 35:100–1. 10.1111/j.1479-828x.1995.tb01844.x 7771984

[62] Rugolo S Mirabella D Palumbo M Chiantello R Fiore G. Complete Wolfram’s syndrome and successful pregnancy. *Eur J Obstet Gynecol Reprod Biol.* (2002) 105:192–3. 10.1016/s0301-2115(02)00150-1 12381487

[63] Kesavadev J Kumar A Shankar A Gopalakrishnan G Permutt M Wasson J An Asian Indian woman with Wolfram syndrome on insulin pump: successful pregnancy and beyond. *Diabetes Technol Ther.* (2011) 13:781–5. 10.1089/dia.2010.0242 21517693

[64] Toppings N McMillan J Au P Suchowersky O Donovan L. Wolfram syndrome: a case report and review of clinical manifestations, genetics pathophysiology, and potential therapies. *Case Rep Endocrinol.* (2018) 2018:9412676. 10.1155/2018/9412676 29850290 PMC5932515

[65] Zhang K Cui X Long Y. Clinical management and obstetric outcome in WFS1 Wolfram syndrome spectrum disorder: a case report and literature review. *Taiwan J Obstet Gynecol.* (2023) 62:440–3. 10.1016/j.tjog.2022.12.011 37188450

[66] Kang H Yang K Kong X. [Genetic and prenatal diagnosis of a Chinese pedigree with autosomal recessive Wolfram syndrome 1 due to compound heterozygous variants of WFS1 gene]. *Zhonghua Yi Xue Yi Chuan Xue Za Zhi.* (2022) 39:698–702. 10.3760/cma.j.cn511374-20210603-00469 35810424

[67] American Diabetes Association Professional Practice Committee. 15. management of diabetes in pregnancy: standards of care in diabetes—2025. *Diabetes Care.* (2025) 48:S306–20. 10.2337/dc25-S015 39651985 PMC11635054

[68] Lucariello A Perna A Sellitto C Baldi A Iannaccone A Cobellis L Modulation of wolframin expression in human placenta during pregnancy: comparison among physiological and pathological states. *Biomed Res Int.* (2014) 2014:985478. 10.1155/2014/985478 24588001 PMC3920918

[69] Nørgaard K Ranjan A Laugesen C Tidemand K Green A Selmer C Glucose monitoring metrics in individuals with type 1 diabetes using different treatment modalities: a real-world observational study. *Diabetes Care.* (2023) 46:1958–64. 10.2337/dc23-1137 37610784

[70] Nimri R Phillip M. Automated insulin delivery systems for treatment of type 1 diabetes: strategies for optimal performance. *Horm Res Paediatr.* (2025) 98:371–83. 10.1159/000543654 39864413

[71] Lee T Collett C Bergford S Hartnell S Scott E Lindsay R Automated insulin delivery in women with pregnancy complicated by type 1 diabetes. *N Engl J Med.* (2023) 389:1566–78. 10.1056/NEJMoa2303911 37796241

[72] Benhalima K Beunen K Van Wilder N Ballaux D Vanhaverbeke G Taes Y Comparing advanced hybrid closed loop therapy and standard insulin therapy in pregnant women with type 1 diabetes (CRISTAL): a parallel-group, open-label, randomised controlled trial. *Lancet Diabetes Endocrinol.* (2024) 12:390–403. 10.1016/S2213-8587(24)00089-5 38697182

[73] Murphy H Roland J Skinner T Simmons D Gurnell E Morrish N Effectiveness of a regional prepregnancy care program in women with type 1 and type 2 diabetes: benefits beyond glycemic control. *Diabetes Care.* (2010) 33:2514–20. 10.2337/dc10-1113 21115765 PMC2992180

[74] Cano A Rouzier C Monnot S Chabrol B Conrath J Lecomte P Identification of novel mutations in WFS1 and genotype-phenotype correlation in Wolfram syndrome. *Am J Med Genet A.* (2007) 143A:1605–12. 10.1002/ajmg.a.31809 17568405

[75] Çelmeli G Türkkahraman D Çürek Y Houghton J Akçurin S Bircan I. Clinical and molecular genetic analysis in three children with wolfram syndrome: a novel WFS1 mutation (c.2534T>A). *J Clin Res Pediatr Endocrinol.* (2017) 9:80–4. 10.4274/jcrpe.2894 27468121 PMC5363171

